# Preliminary Evidence That Design Fluency Is Related to Dual-Task Treadmill Gait Variability in Healthy Adults

**DOI:** 10.3390/neurosci5030026

**Published:** 2024-09-12

**Authors:** Christopher I. Higginson, Morgan K. Bifano, Kelly M. Seymour, Rachel L. Orr, Kurt M. DeGoede, Jill S. Higginson

**Affiliations:** 1 Department of Psychology, Loyola University Maryland, 4501 N. Charles St., Baltimore, MD 21210, USA; bifano@kennedykrieger.org (M.K.B.); drorr@seacoastneuropsych.com (R.L.O.); 2 Department of Mechanical Engineering, University of Delaware, 130 Academy St., Newark, DE 19716, USA; 3 Department of Engineering & Physics, Elizabethtown College, 1 Alpha Dr, Elizabethtown, PA 17022, USA

**Keywords:** gait, dual task, fluency, cognitive flexibility

## Abstract

Evidence supporting a link between gait and cognition is accumulating. However, the relation between executive functioning and spatiotemporal gait parameters has received little attention. This is surprising since these gait variables are related to falls. The goal of this preliminary study was to determine whether performance on measures of inhibition, reasoning, and fluency is related to variability in stride length and step width during dual-task treadmill walking in a sample of healthy adults. Nineteen healthy adults averaging 40 years of age were evaluated. Results indicated that processing speed was reduced, *t*(18) = 6.31, *p* = 0.0001, step width increased, *t*(18) = −8.00, *p* = 0.0001, and stride length decreased, *t*(18) = 3.06, *p* = 0.007, while dual tasking, but variability in gait parameters did not significantly change, consistent with a gait/posture-first approach. As hypothesized, better performance on a visual design fluency task which assesses cognitive flexibility was associated with less dual-task stride length variability, *r_s_*(17) = −0.43, *p* = 0.034, and step width variability, *r* = −0.56, *p* = 0.006. The results extend previous findings with older adults walking over ground and additionally suggest that cognitive flexibility may be important for gait maintenance while dual tasking.

## 1. Introduction

It was once thought that walking was an automatic process in humans that does not require significant thought, as gait was considered largely mediated by the brainstem and spinal cord [1]; however, evidence supporting a link between gait and cognition is accumulating [2]. For example, dual task studies have demonstrated adverse changes in gait and/or cognition when individuals simultaneously walk and perform a cognitive task, suggesting that gait is not automatic [3,4,5]. In addition, gait variability is associated with cognitive decline in otherwise healthy older adults [6,7] and has been correlated with cognitive function and cortical thickness [8], further supporting a link between gait and cognition. 

Various elements of cognition have been studied in relation to gait, but executive functioning has arguably received the most attention. Performance on measures of executive functioning correlates with gait speed in both healthy older adults [9,10,11] and clinical samples [12,13]. Of the various elements of cognition, or cognitive domains, executive functioning is a broad category of thought responsible for guiding behavior; it includes abstract reasoning and inhibition in addition to cognitive flexibility as well as other complex elements of cognition [14]. As the term “executive” suggests, these functions can be viewed as supraordinate to other forms of cognition, directing them to optimize performance under various circumstances.

It seems reasonable that executive functioning is related to gait since this element of cognition is ultimately responsible for all behavioral output, and more specifically, allocating cognitive resources to ongoing tasks while walking. Indeed, executive functioning has been related to gait speed in various samples, as noted above. However, the relation between executive functioning and other elements of gait has received little attention. For example, we are aware of only one study to address the relation between executive functioning and spatiotemporal gait parameters such as stride length and step width [15]. This is surprising since these gait variables are related to falls in older adults [16,17,18,19], a significant public health issue worthy of prevention efforts. In 2020, the medical cost for falls experienced by older adults in the United States was USD 50 billion with over 36,000 older adults dying due to falls [20]. 

In the one study to address this issue, van Iersel et al. [15] observed a significant relationship between performance on measures of executive functioning and dual-task stride length variability. The study used a trail-making task to measure cognitive flexibility [15], a specific element of executive functioning that involves readily alternating one’s mode of thought or action [21]. In trail-making tasks, examinees draw lines to connect points in a specific order, often numerically or alphabetically. A cognitive flexibility component is present when examinees are asked to switch back and forth between the different ordinal schemes. 

The purpose of the current preliminary study was to build on this very limited literature addressing the relation between executive functioning and spatiotemporal gait parameters. We sought to extend the literature into other elements of executive functioning and other spatiotemporal gait parameters. More specifically, our goal was to determine whether performance on measures of inhibition, reasoning, verbal fluency, and design fluency is related to variability in stride length and step width during dual-task treadmill walking in a sample of healthy adults. Stride length variability was considered because it has been related to elements of executive functioning in previous studies, as noted above. Step width variability was considered because several measures of gait variability have been related to falling in previous studies [8]. Regarding executive functioning, measures of inhibition and reasoning were included to extend the literature into these elements of executive functioning. Fluency measures were included because like trail making, they are thought to assess cognitive flexibility [21]. Verbal fluency tasks require examinees to rapidly state words that start with a given letter (a.k.a., orthographic or letter fluency) or belong to a certain category (a.k.a., semantic fluency). 

An especially novel element of the study is the inclusion of a design fluency task. In contrast to verbal fluency, design fluency tasks are visual in nature and have examinees draw as many different designs as possible according to specific rules. All fluency tasks involve executive functioning, and specifically cognitive flexibility, as they require the generation of a plan regarding how to approach this novel task and the flexibility to switch approaches or responses to avoid repetition. Design fluency has been associated with volume of the superior frontal gyrus [22], supporting its interpretation as a measure of the cognitive flexibility element of executive functioning. Design fluency has not been studied in relation to gait but is especially intriguing since cognitive tasks with a visual component are most likely to disrupt gait presumably due to interference with the visuoperceptual resources required for walking [23,24,25,26,27]. 

Based on previous findings that various measures of executive functioning are related to gait variability, we hypothesized that better performance on measures of inhibition, reasoning, and fluency would be significantly correlated with less dual-task stride length and step width variability. By using a dual task paradigm, not only can relations between baseline cognitive function and dual task performance be explored, but changes in gait and cognitive performance when the secondary task is added can also be assessed. Determining which specific cognitive and gait variables are related will hopefully lead to a better understanding of the cognitive contributions to gait and earlier identification of those at risk for cognitive decline or falls, improve fall prevention, and inform rehabilitation efforts.

## 2. Materials and Methods

### 2.1. Participants

A convenience sample of 19 healthy adults (13 female, 17 right-handed) ranging in age from 18 to 80 years were recruited from a university campus community. This sample size affords statistical power adequate to detect medium effects like those found in previous studies [15]. Individuals with a history of muscle or bone injury, head injury or concussion, body mass index > 40, stroke, diagnosed psychological illness (e.g., major depressive disorder, attention-deficit/hyperactivity disorder), significant uncorrected visual deficits, poor balance, or other injuries or conditions that could affect gait or cognition (e.g., heart condition, heart murmur, rapid or irregular heartbeat, chest pain, fainting, dizziness, uncontrolled hypertension, uncontrolled hypercholesterolemia, diabetes, cigarette smoking) were excluded from the study. Participant characteristics are listed in Table 1. This study protocol was approved by the institutional review boards (IRBs) at Loyola University Maryland (HS-2867) and the University of Delaware (188362-12), and written informed consent was obtained from participants prior to data collection.

### 2.2. Procedure

#### 2.2.1. Neuropsychological Measures

Prior to gait analysis, participants completed a battery of cognitive tasks including Design Fluency, Verbal Fluency, and Color-Word Interference Tests from the Delis–Kaplan Executive Function System (D–KEFS) [28], and the Shipley Institute of Living (SILS) Abstraction subtest [29]. 

The Design Fluency Test [28] has several components. In Condition One, examinees connect dots using four straight lines to make as many different designs as possible within 60 s. In Condition Two, the task is repeated, but examinees must connect empty dots and ignore filled dots. In Condition Three, the task is repeated but having to switch back and forth between connecting empty dots and filled dots. The primary outcome score is the total number of unique designs generated in each condition. Because evidence suggests that Conditions One and Two primarily assess fluency and motor planning, while Condition Three primarily assesses visual scanning [30], the number of unique designs generated in Conditions One and Two were added together to create a total Design Fluency score to measure cognitive flexibility. 

Letter Fluency from the Verbal Fluency Test [28] asks examinees to rapidly generate as many words as possible that begin with a specific letter. Names of people, places, and numbers must be avoided, and credit is not given for the same word with a different ending. Three 60 s trails are completed, one trial for each of the letters “F”, “A”, and “S”. The total number of correct words generated was analyzed here as a measure of cognitive flexibility.

The Inhibition condition from the Color-Word Interference Test [28] is a Stroop task. Examinees are presented with a page listing color words that are printed in ink of an incongruent color (e.g., the word “red” printed in blue ink). Examinees are asked to identify the color of the ink in which the words are printed, ignoring the word itself, thereby inhibiting the overlearned response to read the word. Examiners point out any errors as they are made, and examinees are to immediately correct their mistake. Time in seconds to complete the task was analyzed as a measure of inhibition.

The Abstraction subtest from the SILS [29] asks examinees to fill in blanks with individual numbers or letters. For each of 20 items, a sequence of numbers and/or letters is provided, and examinees fill in the blanks to complete the sequence. For example, one item could be “1 2 3 4 5 __” for which the correct response would be “6”. The instructions request that the items be completed in order because they increase in difficulty. Examinees are given 10 min to complete the items, and the raw score is the total number of correct responses multiplied by two. This raw score was analyzed as a measure of abstract reasoning. 

#### 2.2.2. Gait Analysis

Self-selected walking speed was determined by having participants walk at their normal comfortable pace for ten meters over ground. Participants did this twice, and the average time to walk the middle six meters was computed. Next, participants completed 120 s walking trials at their self-selected speed on an instrumented treadmill (Bertec Corporation; Columbus, OH, USA) while an eight-camera passive motion capture system at 60 Hz (Motion Analysis Corporation; Santa Rosa, CA, USA) tracked 48 reflective markers placed on anatomical landmarks. The spatiotemporal variables of stride length and step width were determined, and means and standard deviations were computed. The standard deviations were used to operationalize variability. Cortex (Motion Analysis Corporation; Santa Rosa, CA, USA) and Visual 3D (C-Motion Inc.; Bethesda, MD, USA) were used for data processing. All gait cycles throughout each two-minute trial were included (>100 gait cycles). Kinematic data were filtered using a bi-directional Butterworth low-pass filter at 6 Hz. Walking trials were completed under single task conditions and under dual task conditions in which participants simultaneously walked and completed the Symbol Digit Modalities Test (SDMT) [31] described below. Treadmill walking trials were randomized to address the potential impact of fatigue on performance.

#### 2.2.3. Dual Task

The SDMT [31] is a visual measure of information processing speed in which examinees are presented with a page with a key at the top associating nine simple geometric designs with the numbers one through nine. Below the key are several rows of randomly ordered symbols. Using the key, examinees verbally state the number associated with each symbol in the order presented as quickly as possible for 120 s. Participants completed the SDMT under single task conditions while seated and under dual task conditions while walking. To address practice effects, different versions of the SDMT were completed under single and dual task conditions. The total number of correct responses was recorded as an index of performance.

#### 2.2.4. Statistical Analysis

Initially, Kolmogorov–Smirnov tests were computed to ensure that the variables were normally distributed and parametric statics were appropriate. When variables did not significantly deviate from a normal distribution, Pearson product–moment correlation coefficients were computed to determine the degree of linear association between gait and cognitive variables, and the presence of dual task effects on gait and cognitive variables was determined by computing paired *t*-tests comparing single task and dual task performance [32]. When variables were found to significantly deviate from a normal distribution, nonparametric tests were used. Spearman’s rank correlation coefficient was computed instead of Pearson correlation coefficients, and Wilcoxon signed-rank tests were computed instead of *t*-tests. The personal computer version of the Statistical Package for the Social Sciences (SPSS 22; IBM, Armonk, NY, USA) was used to compute the statistical analyses. Alpha was set to 0.05, and one-tailed analyses were computed because hypotheses were directional. 

## 3. Results

**Figure 1:**
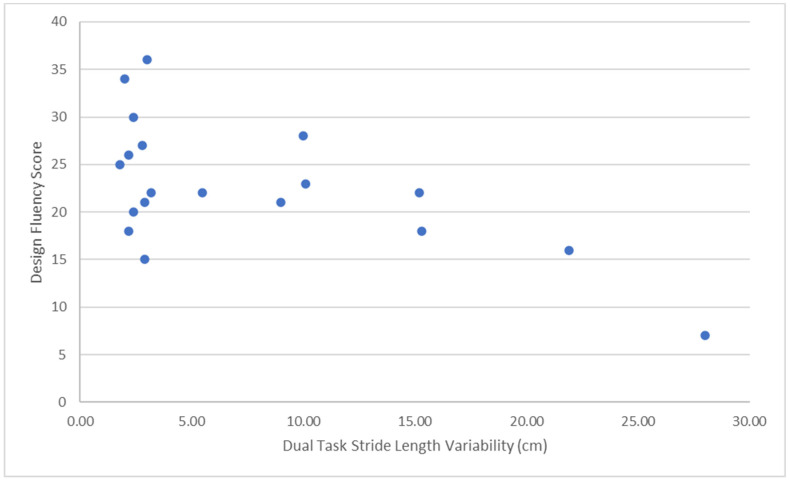
Scatterplot of Design Fluency versus Dual Task Stride Length Variability.

**Figure 2:**
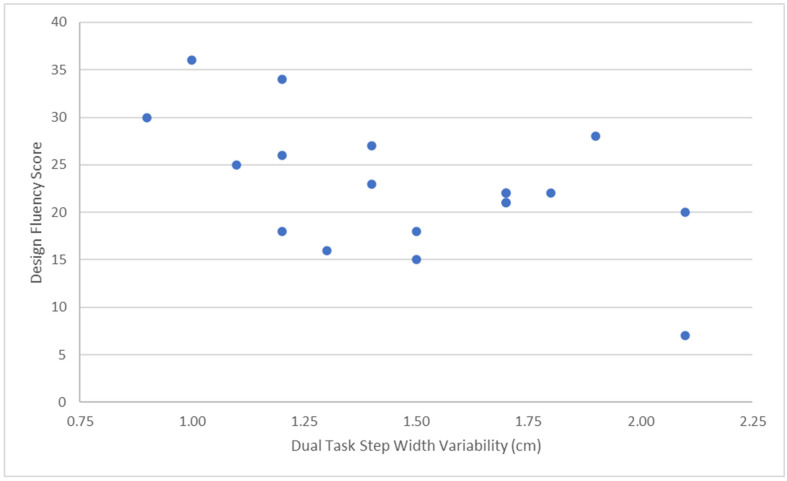
Scatterplot of Design Fluency versus Dual Task Step Width Variability.

Descriptive statistics for single and dual task SDMT, single and dual task spatiotemporal gait parameters, and neuropsychological test raw scores are in Table 2. The Kolmogorov–Smirnov test found that the distributions of three variables significantly deviated from normal, SILS abstraction (*Z* = 0.204, *p* = 0.001), and single and dual task stride length variability (*Z* = 0.264, *p* = 0.001; *Z* = 0.294, *p* < 0.001, respectively). Therefore, Spearman’s rho was computed to assess correlations involving these variables, and the Wilcoxon test was computed to compare related groups. Results of correlational analyses assessing the linear relations between executive functioning and dual task gait variability are in Table 3. Performance on Design Fluency significantly correlated with dual task stride length variability, *r_s_*(17) = −0.43, *p* = 0.034, and step width variability, *r*(17) = −0.56, *p* = 0.006. According to Cohen’s conventions [33], these represent medium to large effects. Figure 1 and Figure 2 depict scatterplots for these statistically significant correlations and illustrate the *r* values are not unduly influenced by outliers. Performance on Letter Fluency did not significantly correlate with stride length variability, *r_s_*(17) = 0.04, *p* = 0.44, or step width variability, *r*(17) = −0.17, *p* = 0.48. Neither Color-Word Interference Test, Inhibition, *r*(17) = 0.04, *p* = 0.89, nor SILS Abstraction, *r_s_*(17) = 0.08, *p* = 0.37, significantly correlated with step width variability. The correlation between performance on Color-Word Interference Test, Inhibition, *r_s_*(17) = 0.22, *p* = 0.18, and stride length variability represented an effect approaching medium size. The correlation between performance on SILS Abstraction and stride length variability was large, *r_s_*(17) = 0.56, but not statistically significant because it was not in the hypothesized direction. In addition, a review of the data suggests that the correlation was influenced by outliers, and the variables do not appear to be linearly related. 

Analyses regarding dual task effects found significant differences between single and dual task performance. Paired *t*-tests revealed significant dual task effects for SDMT raw score, *t*(17) = 5.92, *p* = 0.0001, stride length, *t*(18) = 3.06, *p* = 0.007, and step width, *t*(18) = −8.00, *p* = 0.0001. (Note that single task SDMT score was not available for one participant.) Compared to single task performance, SDMT raw score declined, stride length decreased, and step width increased while dual tasking. Differences between single and dual task performance were not statistically significant for stride length variability, *Z* = −0.58, *p* = 0.56, or step width variability, *t*(18) = 0.46, *p* = 0.92. The data that support the findings of this study are openly available in Mendeley Data at https://doi.org/10.17632/6dc3yb8h5c.1 accessed on 1 July 2024. 

## 4. Discussion

The purpose of the current study was to determine whether performance on several measures of executive functioning related to gait variability during treadmill walking in a sample of healthy adults. Results indicate that performance on a design fluency task, a visual measure of cognitive flexibility, was significantly related to stride length variability and step width variability. The correlations between these variables were medium to large, indicating that better design fluency was associated with less variability, as hypothesized.

This is an important outcome since increased dual task stride length variability and step width variability are related to falling in older adults [16,34,35,36]. In a previous study relating cognitive function to stride length variability, van Iersel et al. [15] reported that a ratio score from a trail-making task reflecting better cognitive flexibility was related to reduced stride length variability in older adults walking over ground. Our results dovetail nicely with those of van Iersel et al. since design fluency has a large cognitive flexibility component; however, our results extend their findings to healthy younger adults and treadmill walking. It appears that the relation between cognitive flexibility and stride length variability is not only present in old age when reduced balance and falls are more common. In addition, the relation is present when individuals are walking on a treadmill, a task that differs from walking over ground [37,38,39].

It seems reasonable that a measure of cognitive flexibility is related to dual-task gait variability. Individuals are presumably shifting their attention between walking and cognition, suggesting a cognitive flexibility component to dual task walking. It also seems reasonable that a task that is visual rather than auditory in nature is related to dual-task gait variability since there is little question that walking has a significant visual component. The cognitive task van Iersel et al. found to correlate with stride length variability, trail making, involves visual stimuli, and the cognitive task performed while walking in this study also involves visual stimuli. 

Although it stands to reason why performance on a measure of design fluency is related to gait variability, it is unclear why performance on measures of other elements of executive functioning did not correlate with gait variability, as such correlations have been observed by others [13,40,41]. One possibility is differences between samples or differences in the exact executive or spatiotemporal variables assessed. Another possibility is that the sample size did not afford adequate statistical power to detect these relationships. Consistent with this, the correlation between performance on a measure of inhibition and stride length variability approached a medium--sized effect but did not reach statistical significance. However, it might simply be the case that dual task walking does not have a significant inhibition component in healthy middle-aged or younger adults. 

Although not directly related to the purpose of the study, results regarding dual task effects include several interesting findings. Regarding spatiotemporal gait variables, step width was increased when simultaneously performing the SDMT. These results are consistent with those of others [27,42] who also reported an increase in step width while dual tasking. As noted by Seymour et al., this may represent a compensatory increase in the base of support due to a feeling of instability, as an increase in step width is characteristic of cautious gait, correlated with fear of falling in older adults [43], and may help to maintain instantaneous lateral stability [44]. 

Stride length was also reduced while performing the SDMT. Shorter stride length while dual tasking has been observed previously in healthy adults [3,24,42], however, the opposite finding of increased stride length has also been reported [45]. One explanation for the discrepant results is that the cognitive task used in the one study found that increased stride length did not involve visual stimuli, but the studies reporting decreased stride length, including this study, used cognitive tasks that were visual in nature [24,42]. Therefore, our dual task effects are consistent with a growing body of evidence that visual cognitive tasks performed while walking lead to gait changes [26,27] and that visual tasks not only affect gait over ground, where visual input is arguably more important due to surface variability, obstacle avoidance, and optic flow, but also affect gait on a treadmill. 

Although stride length was reduced and step width increased while dual tasking, variability in stride length and step width were not significantly different between single and dual task conditions. It appears as though participants made spatiotemporal changes to their gait while dual tasking but remained equally good at regulating and maintaining the modified gait characteristics (i.e., avoided fluctuations) while dual tasking. As noted above, perhaps visual cognitive flexibility is required for gait maintenance while dual tasking, explaining the correlation between visual fluency and dual-task gait variability observed here. In this case, variability did not increase while dual tasking because this sample was healthy and had intact executive functioning.

Performance on the SDMT was worse while walking than while seated. Worse dual task performance on cognitive tasks involving visual stimuli has been reported by others [46]. Increased reaction time [47,48] and marginally reduced inhibition (i.e., Stroop task performance [49]) have also been observed. This overall pattern of dual task results involving cognitive decline, but relatively preserved gait (variability), is consistent with prioritization of gait over cognitive task performance. This “gait/posture first” approach has also been reported by others [50].

Several limitations present in the current study are worthy of note. First, the sample size limited statistical power. To address this issue, we included participants who varied greatly in terms of age. Power appeared to impact the small to medium-sized correlation between performance on a measure of inhibition and stride length variability that was not detected. The limited sample size also prevented multivariate analyses that would better elucidate the relative importance of the various cognitive tasks in predicting the gait variables. Second, the single task condition for the SDMT was completed while seated. Ideally, this task would have been completed while standing to reduce discrepancy between single task and dual task (walking) conditions. Therefore, it remains possible that changes in performance on the SDMT between single and dual task conditions were due to standing rather than walking. Third, the sample consisted of healthy adults who are not at great risk of falling. However, this can equally be viewed as a strength as it extends the literature to younger adults and speaks to the nature of the relations between gait and cognition in individuals with relatively good balance. 

In summary, these results suggest that Design Fluency is related to gait variability while dual tasking. As hypothesized, better fluency was related to less variability in both stride length and step width, gait characteristics associated with falling risk. The results support a growing body of literature indicating a relationship between executive functioning and gait, and extend the literature by providing initial, preliminary evidence that visual cognitive flexibility is related to gait variability during dual task treadmill walking in a sample of healthy adults ranging in age. These preliminary results have both clinical and experimental implications. They suggest that visual cognitive flexibility may play a role in gait in healthy adults. This seems reasonable since when humans walk, we frequently must shift our attention alternately from the tasks of safely walking to the various cognitive and motor tasks that we perform simultaneously. The results are also consistent with other data suggesting that healthy adults tend to adapt their gait to a more cautious style while dual tasking, and this adaptation is successful insomuch as it does not result in increased gait variability but comes at the cost of reduced cognitive processing. This “gait first” approach makes sense since it prioritizes safety. The results also suggest that assessment of visual cognitive flexibility may help to identify individuals at risk of falling and rehabilitation efforts to improve cognitive flexibility may reduce falling risk. From a research perspective, these preliminary results suggest that the relationship between visual cognitive flexibility (and perhaps other less common measures of executive functioning) and gait variability warrants further attention. Future studies should attempt to replicate these findings. They should do so in larger samples with specific age groups so that multivariate analyses can be utilized. The results should also be replicated in clinical samples of individuals at risk for falling, such as individuals with a movement disorder such as Parkinson’s disease or individuals with a form of dementia such as Alzheimer’s disease. Future studies could also determine whether changes in visual cognitive flexibility are predictive of later gait changes or whether rehabilitation interventions addressing visual cognitive flexibility lead to changes in spatiotemporal gait parameters and reduced risk of falling.

## Figures and Tables

**Table 1:** Participant Characteristics.

	Mean	*SD*
Age (years)	39.74	22.65
Education (years)	14.74	2.88
BMI (kg/m^2^)	24.76	4.19
Walking Speed (m/s)	1.10	0.21

*Note.* BMI = Body Mass Index.

**Table 2:** Descriptive Statistics for Executive Functioning and Gait Variables.

		Mean	Median	*SD*	IQR
Executive Measures					
	Design Fluency	22.68		6.79	
	Verbal Fluency	43.11		11.21	
	Inhibition	46.58		9.83	
	Abstraction		34		8
Single Task Variables					
	Symbol Digit Modalities Test	84.56		15.83	
	Stride Length (cm)	123.1		17.67	
	Stride Length Variability (cm)		3.6		5.8
	Step Width (cm)	16.58		1.65	
	Step Width Variability (cm)	1.48		0.41	
Dual Task Variables					
	Symbol Digit Modalities Test	66.95 ***		14.60	
	Stride Length (cm)	120.42 **		17.27	
	Stride Length Variability (cm)		3.0		7.7
	Step Width (cm)	18.94 ***		2.25	
	Step Width Variability (cm)	1.50		0.35	

*Note.* IQR = Interquartile Range. ** *p* < 0.01. *** *p* < 0.001.

**Table 3:** Correlations Between Executive Functioning Measures and Dual Task Gait Variability.

	Stride Length Variability	Step Width Variability
Design Fluency	−0.43 *	−0.56 **
Verbal Fluency	0.04	−0.17
Inhibition	0.22	0.04
Abstraction	0.56	0.12

* *p* < 0.05. ** *p* < 0.01.

## Data Availability

The data that support the findings of this study are openly available in Mendeley Data at https://doi.org/10.17632/6dc3yb8h5c.1 accessed on 1 July 2024.
